# Using Green, Economical, Efficient Two-Dimensional (2D) Talc Nanosheets as Lubricant Additives under Harsh Conditions

**DOI:** 10.3390/nano12101666

**Published:** 2022-05-13

**Authors:** Jun Zhao, Tong Gao, Jie Dang, Weiyu Cao, Ziqi Wang, Shuangxi Li, Yijun Shi

**Affiliations:** 1 College of Mechanical and Electrical Engineering, Beijing University of Chemical Technology, Beijing 100029, China; zhaojun@mail.buct.edu.cn (J.Z.); bjhggt@126.com (T.G.); dangjie@buct.edu.cn (J.D.); gaoxingwangziqi@126.com (Z.W.); 2 Division of Machine Elements, Luleå University of Technology, 97187 Luleå, Sweden; 3 State Key Laboratory of Organic-Inorganic Composites, Beijing University of Chemical Technology, Beijing 100029, China; caowy@mail.buct.edu.cn

**Keywords:** 2D nanomaterial, additive, grease, green lubrication, friction, wear

## Abstract

Two-dimensional (2D) nanomaterials have attracted much attention for lubrication enhancement of grease. It is difficult to disperse nanosheets in viscous grease and the lubrication performances of grease under harsh conditions urgently need to be improved. In this study, the 2D talc nanosheets are modified by a silane coupling agent with the assistance of high-energy ball milling, which can stably disperse in grease. The thickness and size of the talc nanosheet are about 20 nm and 2 µm. The silane coupling agent is successfully grafted on the surface of talc. Using the modified-talc nanosheet, the coefficient of friction and wear depth can be reduced by 40% and 66% under high temperature (150 °C) and high load (3.5 GPa), respectively. The enhancement of the lubrication and anti-wear performance is attributed to the boundary adsorbed tribofilm of talc achieving a repairing effect of the friction interfaces, the repairing effect of talc on the friction interfaces. This work provides green, economical guidance for developing natural lubricant additives and has great potential in sustainable lubrication.

## 1. Introduction

With the development of advanced machines, the working conditions of machines become more and more complicated, due to extreme pressure, high temperature, irradiation [1,2,3,4,5,6,7], etc. Friction and wear are the main reasons for the machine failure. Using lubricants is one of the most widely used strategies for friction and wear reduction [8,9,10,11]. Hence, it is of great importance to develop high-efficiency lubricants. Grease is always used in harsh working conditions, but the performance of grease is greatly affected by temperature and load [12]. At high temperatures, grease is usually oxidized to generate unwanted compounds, which damage the lubrication performance, or even leads to the failure of lubrication [13].

Although lubricating additives takes only a small proportion of grease, the tribological properties of grease depend on the additives to a great extent [13]. Hence, it is of great importance to develop novel additives with excellent performance for greases under harsh conditions. In recent years, nanomaterials as lubricant additives have drawn attention from a large number of researchers, due to their excellent antifriction and anti-wear performance [14,15]. Nanoparticles such as Cu, Fe_3_O_4_, and TiO_2_ can effectively reduce friction and wear, especially under boundary lubrication regimes [14,15,16,17]. Two-dimensional (2D) nanomaterials, due to their excellent self-lubricating properties, have attracted much attention [18,19,20]. The typical two-dimensional (2D) nano additives are MoS_2_, WS_2_, graphene, talc, etc. These 2D materials, with one or a few atomic layers, have excellent tribological properties [21,22,23,24]. Because of the high surface energy, nanomaterials usually show a strong tendency to aggregate in lubricants [1,25,26]. In addition, the additive will be recognized as ecofriendly additives for anti-friction and/or anti-wear in lubricating systems if the additive will not release SAPS (sulfated ash, phosphorous and sulfur; SAPS would cause air pollution such as acid rain and haze weather) [27,28,29,30]. Thus, green nanomaterials have been considered as the promising alternative to the typical additive of zinc dialkyl dithiophosphates (ZDDPs), which contain phosphorus and sulfur.

Talc is a 2D layered and naturally abundant mineral; thus, it is low cost, ecofriendly, and stable [31,32]. Its specific gravity ranges from 2.7 to 2.8, and it offers high chemical inertness. Talc layers are weakly bonded with each other through Van der-Waals forces forming the lamellar structure, which facilitates easy shearing, giving it a good self-lubrication performance because the lamellar structure facilitates easy shearing [32,33,34]. Therefore, 2D talc nanosheets are considered as one of the best candidate materials for the development of green lubricating additives. Talc is widely used as solid lubricant with the properties of high crystallinity, low electrical conductivity, high thermal stability and good adsorption properties. It has been found that using talc as lubricant additives can enhance the lubrication of commercial engine oil. Talc also shows potential as an eco-friendly extreme pressure lubricating additive [31,32]. However, it is difficult to disperse talc nanomaterials in viscous grease, and their enhancement for the lubrication of grease under harsh conditions is unknown.

In this study, to improve the dispersion of talc in grease, 2D talc nanosheets are prepared via high-energy ball milling with the assistance of a silane coupling agent. The modified talc can be stably dispersed in grease and has much better tribological properties than those of non-modified talc. The micro structures are characterized and the lubrication performances are studied under harsh conditions. The lubrication mechanism has also been discussed. This work provides green, economical guidance for developing natural lubricant additives and has great potential in sustainable lubrication.

## 2. Experimental

### 2.1. Raw Materials and Instruments

The commercial talc, noted as unmodified talc was purchased from Shanghai Yuanjiang Chemical Co., Ltd. (Shanghai, China). Lithium-based lubricating grease is number 0 of Shangbo, noted as base grease was from Sinopec Lubricating Oil Co., Ltd. (Beijing, China). The silane coupling agent (KH550) came from Nanjing Daoning Chemical Co., Ltd. (Nanjing, China). All materials were the analysis reagents. The ball crusher for preparing the samples was from Changsha Miqi Technology Manufacturing Co, Ltd. (Changsha, China). The friction tests were conducted on the SRV-4 tester (Optimal Instruments, Schwabisch Hall, Germany). The friction pairs were in a ball–disc contact form. The material of both the upper and lower parts of the friction pair was bearing steel (GCr15) with a roughness of 20 nm and a hardness of 650–700 HV. To study the influence of temperature on the tribological properties of talc-grease grease, the temperature was varied from 80 °C to 175 °C under 150 N. To study the influence of load on the tribological properties of talc-grease grease, the load was varied from 150 N to 550 N under 80 °C. The experiments were repeated three times for each condition. The friction conditions were summarized in Table 1. The morphologies of the nanosheets were characterized by scanning electron microscopy (SEM; FEI Quanta 200 FEG, Eindhoven, The Netherlands) and high-resolution transmission electron microscopy (TEM; JEM-2010, Tokyo, Japan) with an accelerating voltage of 120 kV. The surface elements were determined by energy dispersive spectroscopy (EDS; Oxford X-MaxN, Oxford, UK) with an accelerating voltage of 15 kV. The crystal lattice structure and the orientation were identified by X-ray diffraction (XRD) with a Bruker D8 Advance diffractometer (Bruker, Billerica, MA, USA). The chemical structure was obtained by a Fourier Transform Infrared Spectroscopy (FTIR, Vertex 70 V, NETZSCH, Selb, Germany). Three-dimensional (3D) White-light interferometry microscopy (Nexview, ZYGO Lamda, Middleton, CT, USA) was used to detect the widths and depths of wear tracks.

### 2.2. Preparation of Grease

**Figure 1:**
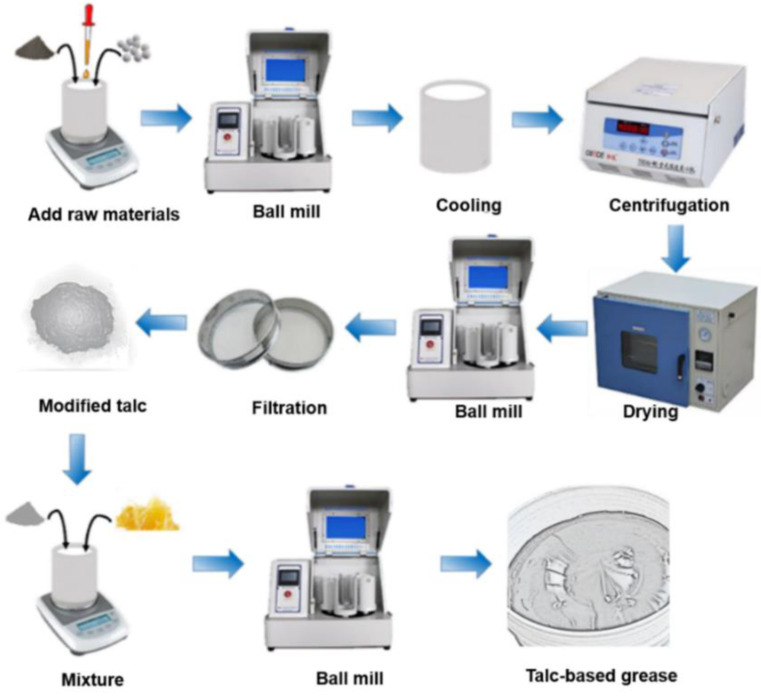
Preparation flowchart of talc-based grease.

Mechanochemical method from ball milling is by virtue of mechanical energy to induce chemical reactions for preparing new materials or modifying materials [35,36,37]. In this experiment, a silane coupling agent was selected as the modifier of talc powder during ball milling processing. As shown in Figure 1, talc powder and the silane coupling agent were weighed as 1:1, and placed in the ball-grinding tank. The mass ratio of grinding the ball (Φ 4 mm) to talc nanosheets was 50:1. The speed of the ball mill was 400 r/min. The mill time was 4 h for the full reaction between the talc and the silane coupling agent. After cooling to room temperature, the samples were washed three times with acetone centrifugation. The centrifuge was rotated at 5000 r/min for 5 min. Filtering was then performed after the centrifugation. The modified talc was put in a vacuum chamber at 50 °C for 1 h. After that, the modified talc was added to the grease. The mass ratio of milling ball to the talc/grease mixture was 5:1. The speed of the ball mill process had a speed of 200 r/min for 4 h. After filtering, the prepared, modified talc-based grease was achieved. Via the same ball mill process without the agent, the unmodified talc-based grease was also obtained.

## 3. Results and Discussion

### 3.1. Characterization of Talc

**Figure 2:**
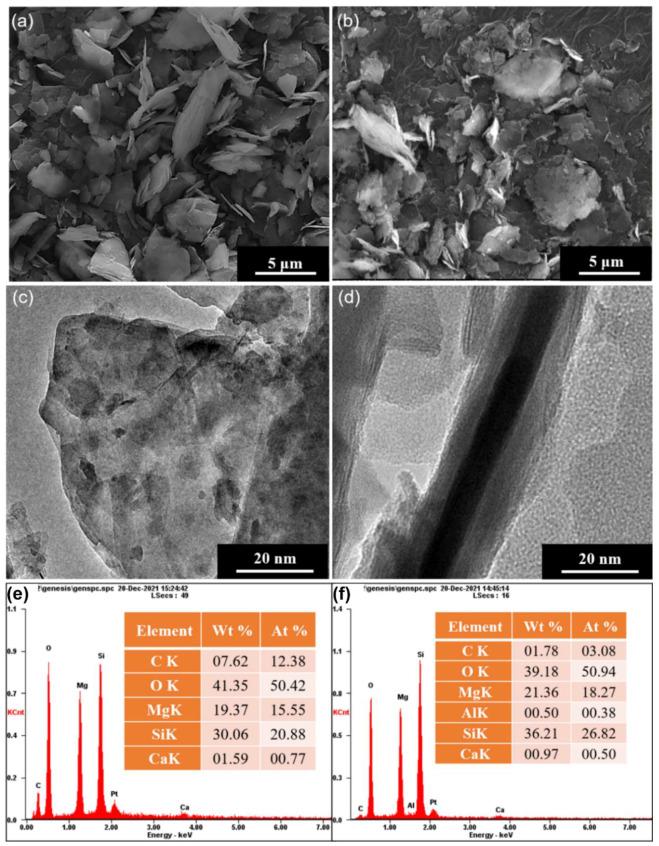
Characterization of talc nanosheets: SEM morphologies of unmodified talc (**a**) and modified talc (**b**); TEM morphologies of modified talc (**c**,**d**); EDS element analysis of the unmodified talc (**e**) and modified talc (**f**).

The morphological characteristics of the unmodified talc and modified talc are well characterized by SEM and TEM. As shown in Figure 2, the unmodified talc exhibits an obvious 2D layered structure, which is uniformly distributed within the range of about 2–3 μm. There are some wrinkled and exfoliated structures obtained for the talc via ball milling (Figure 2b). From the TEM image (Figure 2c,d), the talc shows relatively integrated lamellar structure and the layer thickness is about 20 nm. According to the EDS analysis (Figure 2e,f), the modified talc with the silane coupling agent has a higher fraction of Si than that of unmodified talc, which means that the agent has successfully grafted to the talc surface.

**Figure 3:**
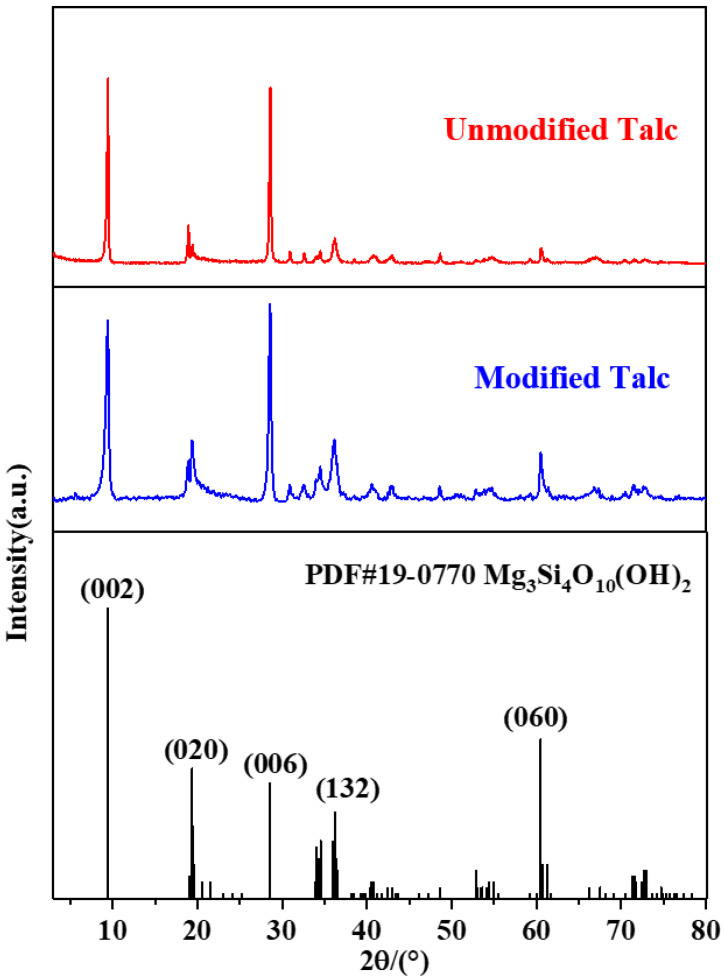
XRD pattern of talc nanosheets and standard spectra of the talc (Mg_3_Si_4_O_11_(OH)_2_).

Figure 3 shows the XRD pattern of the talc powder. It can be seen that the characteristic diffraction peaks of the modified talc and the unmodified talc are consistent with that of the standard talc (Mg_3_Si_4_O_10_(OH)_2_) spectrum, i.e., 9.45°, 19.32°, 28.59°,36.21°, 60.50° and 59.937° [38]. Thus, the modified talc keeps an ordered crystal structure. The undamaged 2D structure of the talc with easy shearing effect will make a great contribution to lubrication performance.

**Figure 4:**
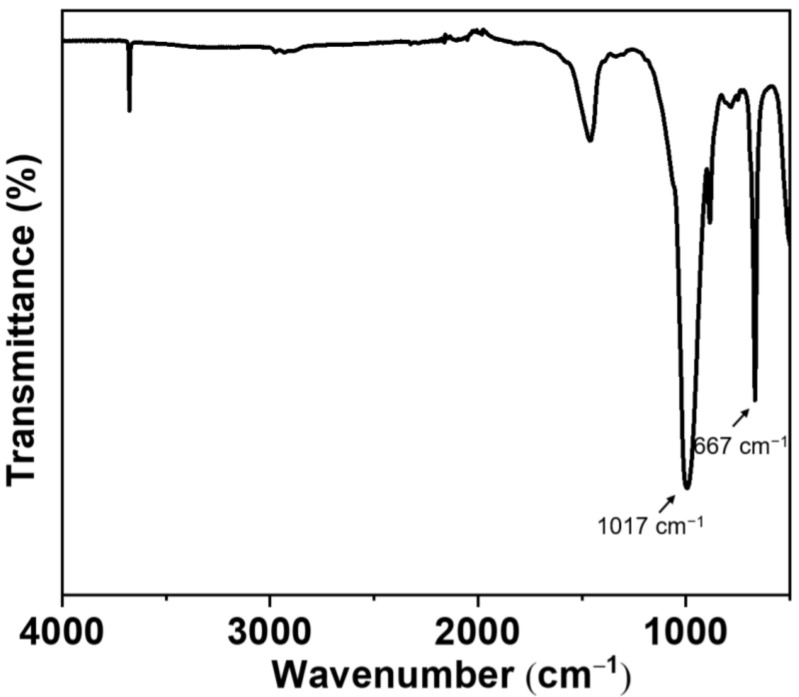
FTIR analysis of the modified talc nanosheet.

In addition, the chemical structure of the modified talc is analyzed by FTIR (Figure 4). The modified talc shows the absorption peak at 667 cm^−1^, which is resulted from MgO (Mg=O stretching) in talc [39]. The obvious absorption peak of Si–O–Si stretching at 1017 cm^−1^ means the talc surface is successfully modified by the agent via a covalent bond [40,41]. Thus, the dispersion and lubrication performance of the nanosheets in grease have a great potential to be realized.

**Figure 5:**
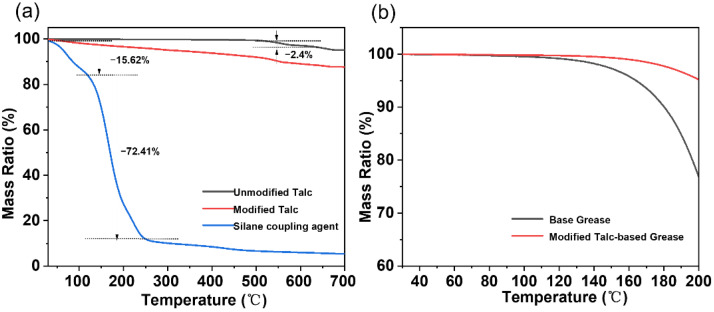
The thermal stability of the talc nanosheets (**a**) and grease (**b**).

The results of TG curves of talc and grease are shown in Figure 5. The temperature ranges from 30 °C to 700 °C with a heating rate of 10 °C/min in nitrogen environment. Compared with the unmodified talc and the agent, it can be concluded that the weight loss of the modified talc (5%) within the temperature range between 30 and 180 °C results from the release of the grafted agents. The weight loss of unmodified talc and modified talc are only 2.4% and 8.9%, respectively, until about 500 °C, indicating the talc nanosheet is very thermostable. This is one reason that the talc improves the thermal stability of grease as shown in Figure 5b.

### 3.2. Lubrication Performance

**Figure 6:**
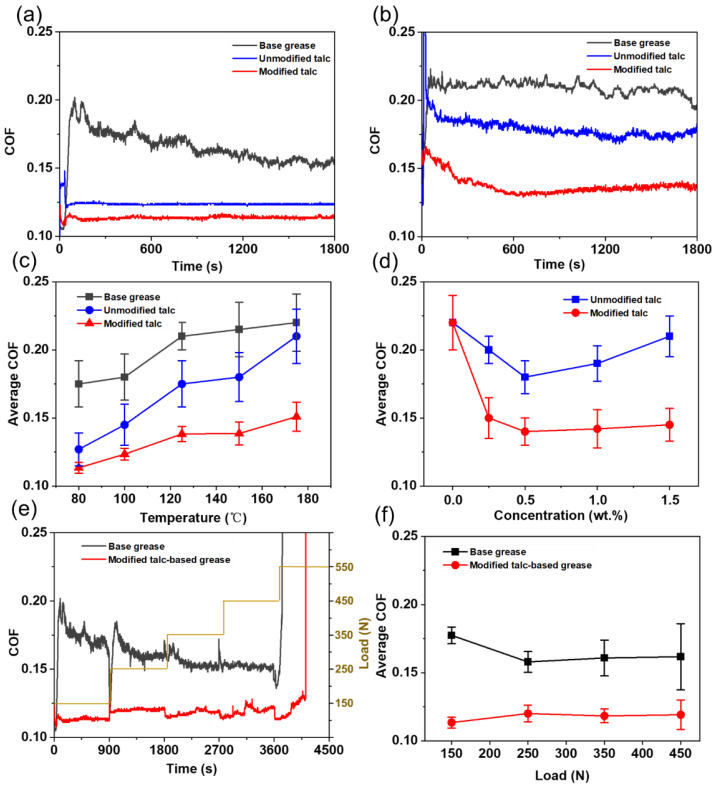
COFs of talc-based grease compared with base grease at the temperature of 80 °C (**a**) and 150°C (**b**); the COFs at different temperature (0.5 wt.%) (**c**) and at different concentration (150 °C) (**d**) under the load of 150 N; the effect of load on the lubrication (**e**,**f**) at the temperature of 80 °C.

Friction from the moving machine pairs generates a lot of heat and the local instantaneous temperature can reach as high as 300 °C [14]. The high-temperature tribological tests are more representative of the actual operating conditions in industrial machinery. So it is crucial to study the effect of high temperature on lubrication properties of grease. It can be seen from Figure 6a–c that the COF of modified talc-based grease (0.5 wt.%) is always stable and lower at high temperature compared with the one without talc. Due to the poor fluidity and insufficient thermal stability of grease, the COF of base grease fluctuates violently and it increases obviously when the temperature is higher than 100 °C. It can be dramatically decreased by adding the modified talc nanosheets at a low concentration of 0.25 wt.%. The optimized concentration is about 0.5 wt.% (Figure 6d), where the COF can be decreased by about 40%. For the unmodified talc-based grease, the lubrication performance is not good and the COF is as high as 0.18. It confirms that the modified talc has a better lubrication performance. The load conditions also have a significant influence on the lubrication performance (Figure 6e,f). It can be seen that under the load varying from 150 N to 450 N, the modified talc-based grease maintains a relatively stable average COF (0.11–0.13), whereas the average COF of base grease is always higher than 0.15. Although the lubrication performance fails when the load increases to 550 N, the modified talc-based grease keeps a relatively longer stability than that of base grease.

### 3.3. Anti-Wear Performance

**Figure 7:**
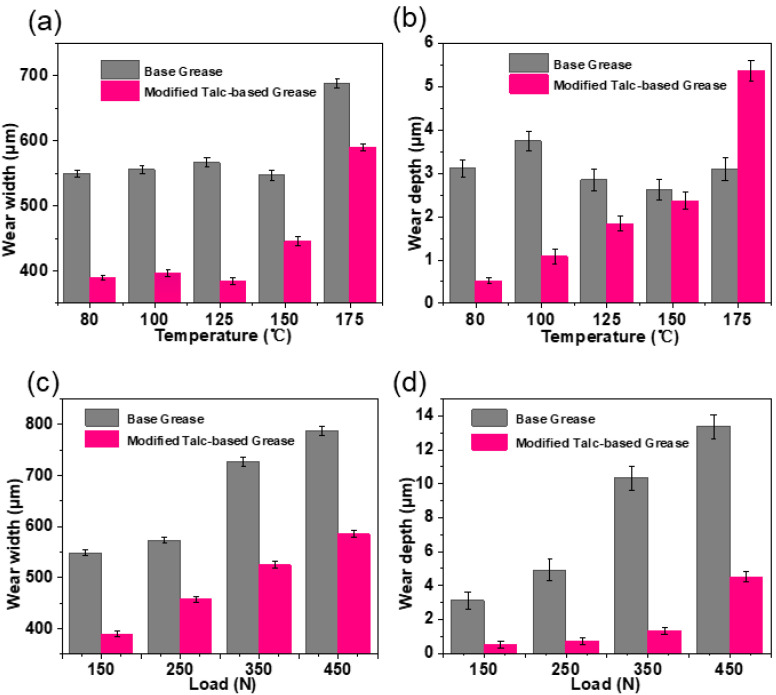
The anti-wear performance of the modified talc-based grease (0.5 wt.%): wear width (**a**) and wear depth (**b**) at different temperature (150 N); wear width (**c**) and wear depth (**d**) under different loads (80 °C).

After the friction tests, the wear scars on the steel disc were successively investigated to evaluate the anti-wear effect of modified talc nanosheets. It can be seen that the wear width and wear depth of the disc lubricated by modified talc-based grease are much smaller than those of base grease even at the high temperature of 150 °C (Figure 7a,b). The wear behavior for base greases is worse when the temperature is 175 °C.

It is because temperature has a crucial effect on the viscosity of grease. At high temperature, the bearing capacity of grease film decreases, and results in severe direct contact of interfacial asperities, leading to increase in friction. However, because of the modified talc-based protective film formed on the rubbing surface, the modified talc-based grease exhibits a significant lubrication performance [13,42]. Although the wear width and wear depth increase with the increase in load, the modified talc-based grease exhibits a better anti-wear performance under different loads. The wear width and wear depth can be reduced by 26% and 66% under the load of 450 N (maximum contact stress: 3.5 GPa) (Figure 7c,d).

**Figure 8:**
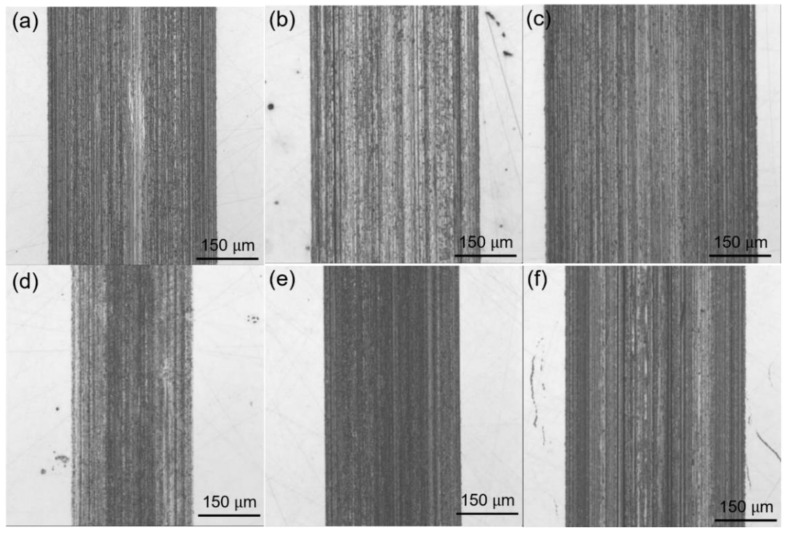
Optical microscope images of wear tracks at different temperature (150 N): base grease at 80 °C (**a**), 150 °C (**b**) and 175 °C (**c**); modified talc-based grease at 80 °C (**d**), 150 °C (**e**) and 175 °C (**f**).

**Figure 9:**
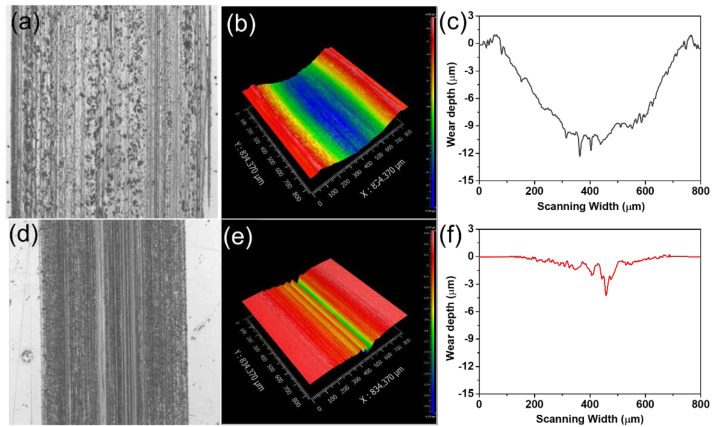
Optical microscope images, 3D morphology and cross section of the wear tracks on the steel disc lubricated by base grease (**a**–**c**) and the modified talc-based grease (**d**–**f**) under the high load of 450 N (80 °C).

The morphologies of the wear scars on the disc are characterized with an optical microscopy (Figure 8). The addition of modified talc can effectively reduce the geometric size of wear scar and improve the anti-wear performance of the grease. At a high temperature (125 °C), several furrows appear on the surface of discs lubricated by base grease. For comparison, there is no visible furrow mark when the added talc nanosheets concentration is 0.5 wt.%. Thus, it is confirmed that the modified talc nanosheets are able to enhance the grease performance at high temperature. In addition, in the case of base grease under high load (3.5 GPa), deep and wide wear scars are observed on the steel disc and the depths of the wear scar for the lubrication of base grease reach 13.36 µm (Figure 9a–c). These results suggest that base grease could not provide good anti-wear performance, and could not protect the friction pair well in practical applications. In comparison, the rubbing surface lubricated by the nanosheets is very smooth under the load of 150 N (2.5 GPa). Although the wear depth and wear width increase at a higher load (3.5 GPa), the anti-wear performance of the nanosheets-filled grease under a high load is much better than that of unfilled grease. These results indicate that the modified talc nanosheet could provide an excellent anti-wear performance (Figure 9d–f).

**Figure 10:**
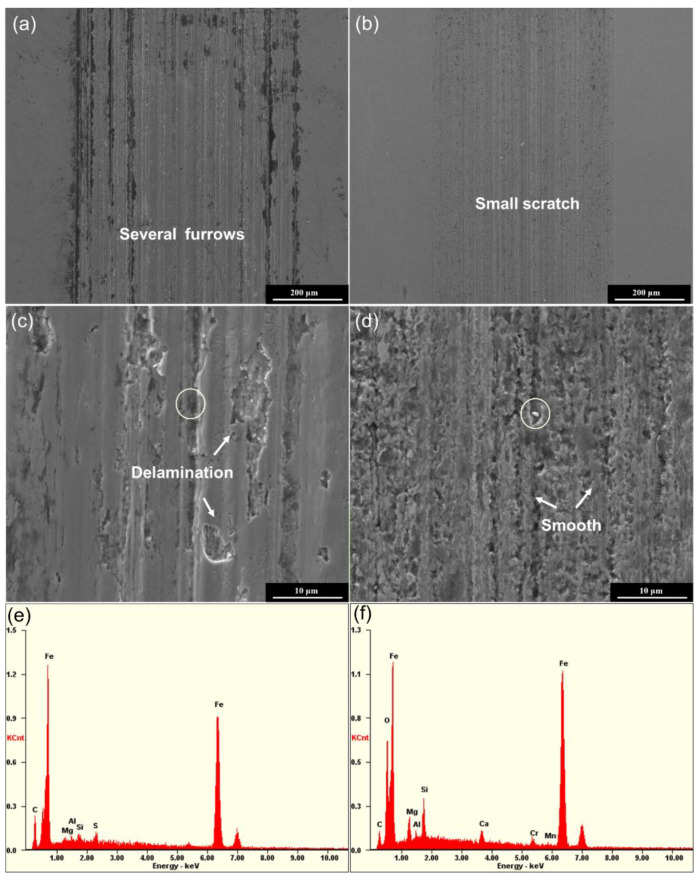
SEM images of wear tracks after the friction test of base grease (**a,c**) and the modified talc-based grease (**b**,**d**); EDS analysis of the wear tracks tested from the base grease (**e**) and the modified talc-based grease (**f**).

The typical SEM morphologies of the worn surfaces lubricated by base grease exhibits long wear tracks, as shown in Figure 10a, because worn surface asperities between the friction pairs could directly contact with each other, and scratch the friction interfaces during the boundary lubrication. The worn surface shows obvious abrasive and delamination wear (Figure 10c). Figure 10b,d show some slight scratches and tracks on the surface lubricated by the nanosheet-based grease. The above results agree with the result of optical morphologies of wear tracks in Figure 9. It can be seen that there are main Fe, Cr, C and O elements in or out of the wear area under the lubrication of base grease. For comparison, there is some residual talc on the cleaned surface, which is identified according to obvious Mg and Si elements appearing on the talc-lubricating surface, i.e., it results from the talc (Mg_3_Si_4_O_10_(OH)_2_) adsorbed on the rubbing surface. It means that the talc is able to form a protective boundary film and repair the rubbing surface to enhance the lubrication performance. Easy layer-shearing effects and the thermostability of talc further improve the lubrication performance [31,32]. On the basis of the tribological analysis described above, as well as the convenient and economic modification route of the talc, the modified talc is confirmed as a promising additive for efficient and green lubrication.

## 4. Conclusions

The 2D talc nanosheets were modified by a silane coupling agent with the assistance of highly-energy ball milling. The effect of talc nanosheets on the lubrication performance of grease was studied using a SRV-4 tribometer. The micromorphology of the modified talc was characterized by SEM, EDS, and TEM methods, together with XRD, FTIR and TG. In addition, the lubrication performance of grease was investigated in detail. The conclusions achieved are as follows:

(1) The silane coupling agent was successfully coupled with the talc nanosheets via ball milling. The modified talc nanosheet has a non-defected crystal structure with a thickness and size of about 20 nm and 2 µm.
(2) The modified talc has a much better lubrication performance than the non-modified talc and base grease. The optimum addition level of modified talc is 0.5 wt.%. The modified talc can greatly enhance the lubrication performance under high temperature and high load. Compared with the base grease, the coefficient of friction and wear depth can be reduced by 40% and 66% at high temperature (150 °C) and high load (3.5 GPa), respectively.
(3) The modified talc nanosheets via ball milling together with grafting of the agent can be uniformly dispersed in viscous grease, which has a good chance to make the nanosheets enter the wear interface quickly and form a protective adsorbed tribofilm. This study provides a green and economical way for using nanomaterial as an efficient lubricant additive and has great potential for application.

## Figures and Tables

**Table 1:** Experimental conditions for study on the lubrication performance of grease.

Experimental Conditions	Parameter
Temperature	80–175 °C
Normal load	150–550 N (2.5–3.5 Gpa)
Sliding stroke	2 mm
Frequency	20 Hz
Sliding time	30 min
Relative humidity	50%
Test number	3

## Data Availability

The data used to support the findings of this study are available subject to approval from the relevant departments through the corresponding author upon request.
